# Supplementary figures and images for: The cervicovaginal microbiome associates with spatially restricted host transcriptional signatures throughout the human ectocervical epithelium and submucosa
Source: PLoS Pathog. 2025 Nov 11;21(11):e1013677. doi: 10.1371/journal.ppat.1013677 (PMC12626330; doi:10.1371/journal.ppat.1013677)

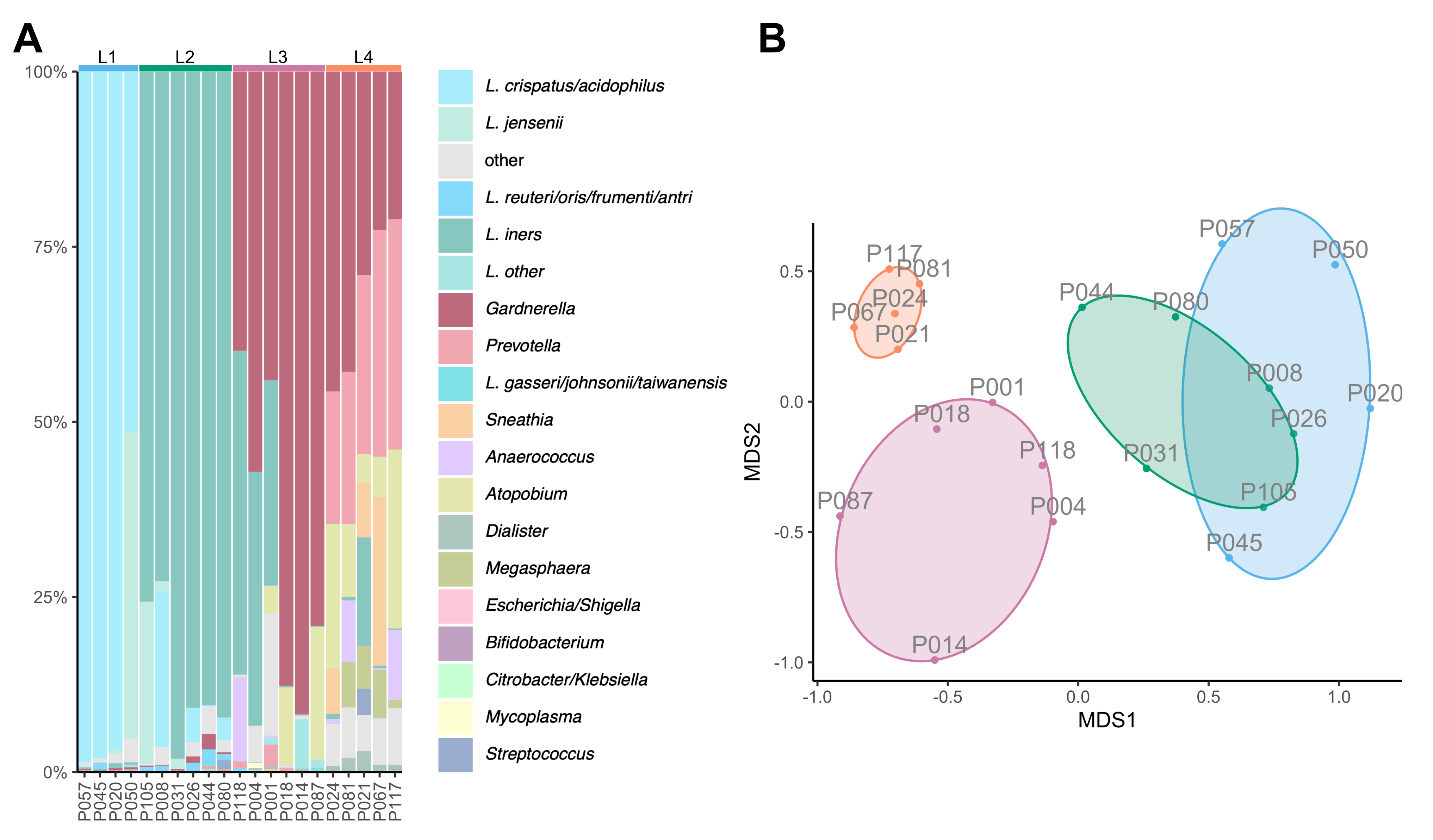

Supplement: S1 Fig — A Bar plot of 16S taxonomical relative abundance for each sample, ordered by microbial groups L1-L4. B Non-metric MultiDimensional Scaling (NMDS) of normalized taxonomic counts. Ellipses encircle the samples belonging to the four microbiome groups L1 (blue), L2 (green), L3 (pink) and L4 (orange) respectively. (TIFF) [file ppat.1013677.s001.tiff]

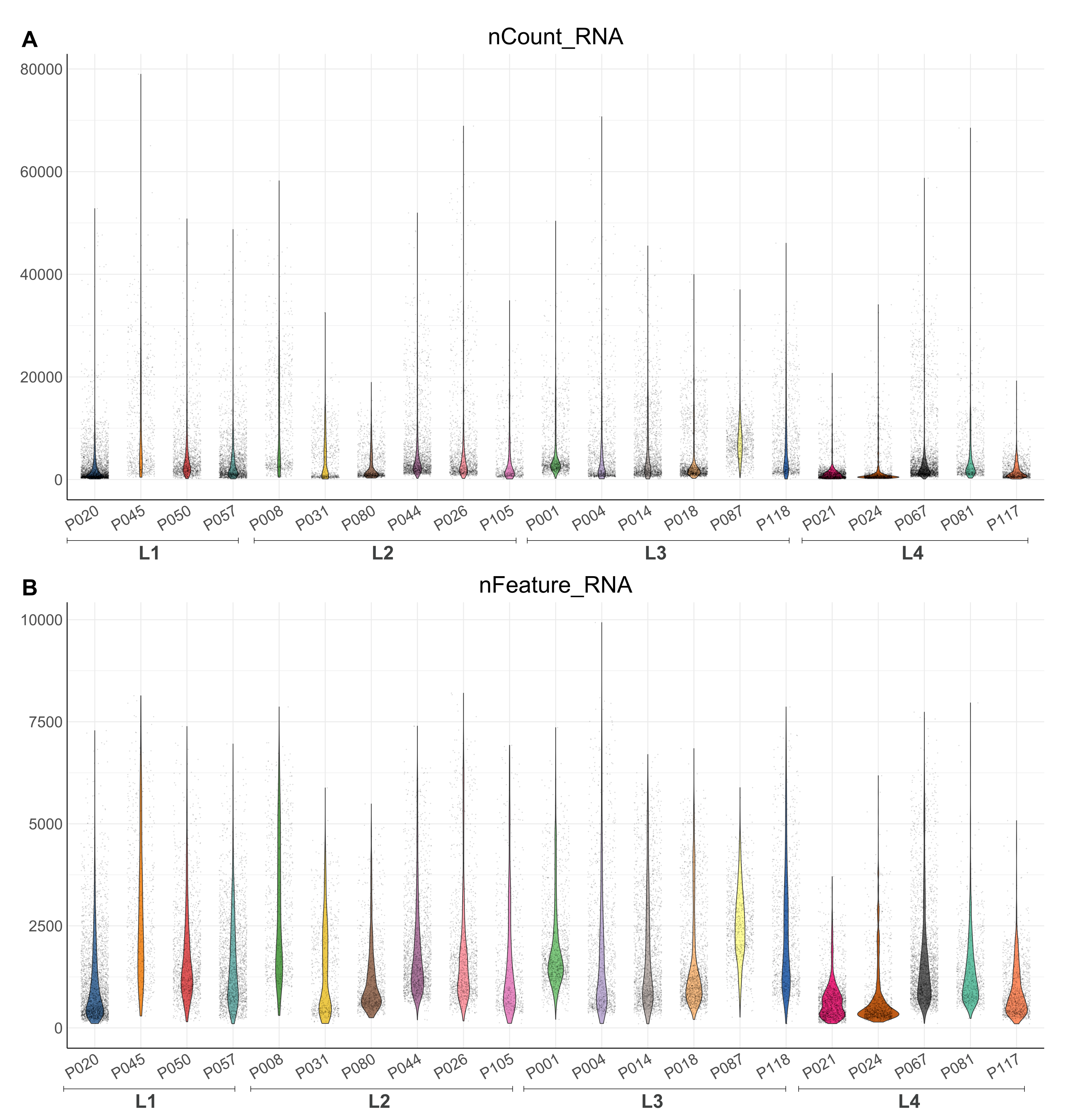

Supplement: S2 Fig — Violin plots of total transcripts/counts A and genes/features B for individual samples. The study groups exhibited the following distribution of total counts/transcripts: (median: L1: 2,176; L2: 2,884; L3: 3,226; L4: 1,422) and genes/features (median: L1: 1,237; L2: 1,438; L3: 1,601; L4: 863). (TIFF) [file ppat.1013677.s002.tiff]

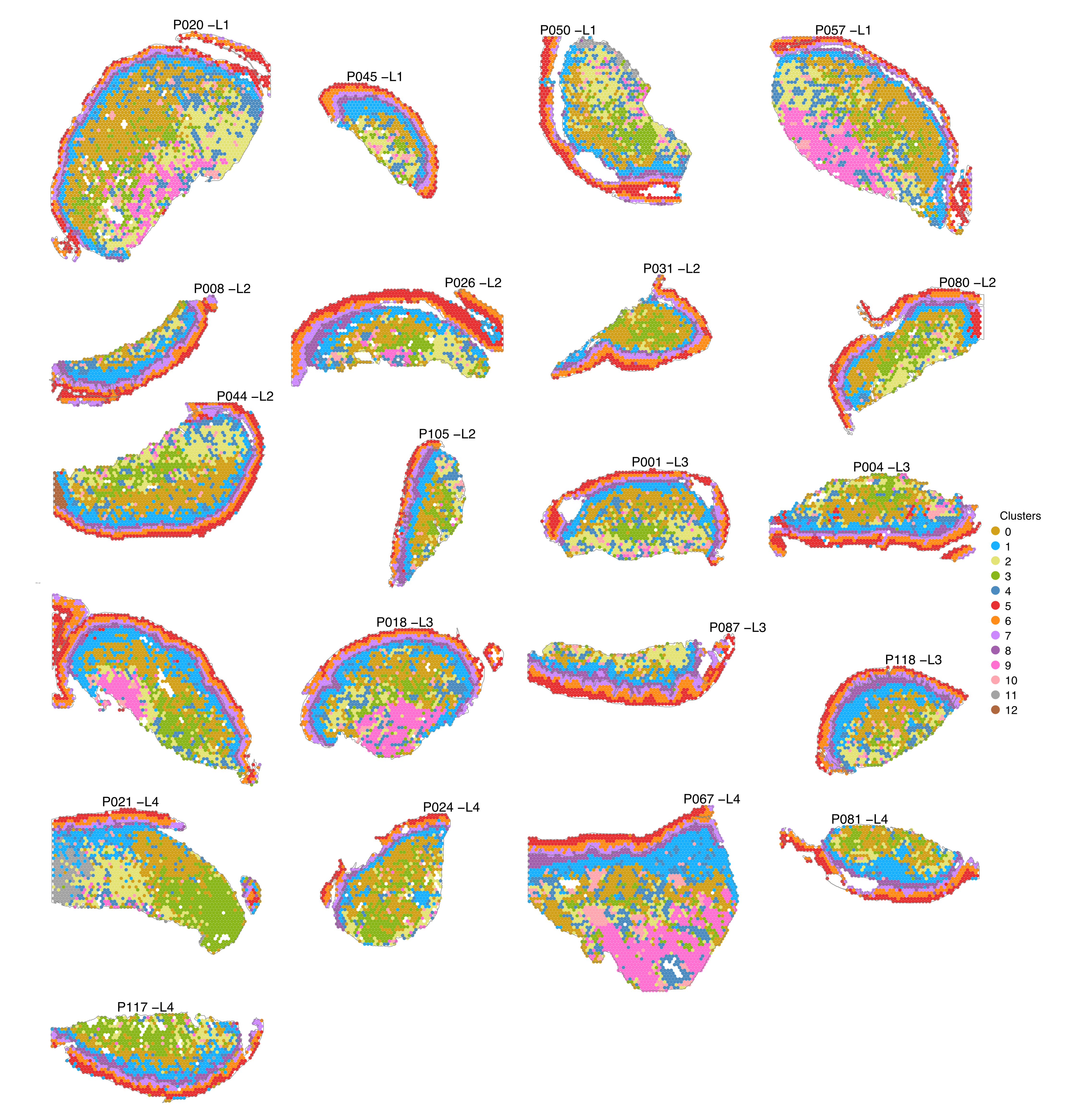

Supplement: S3 Fig — Spots colored by unsupervised Louvain clustering. (TIFF) [file ppat.1013677.s003.tiff]

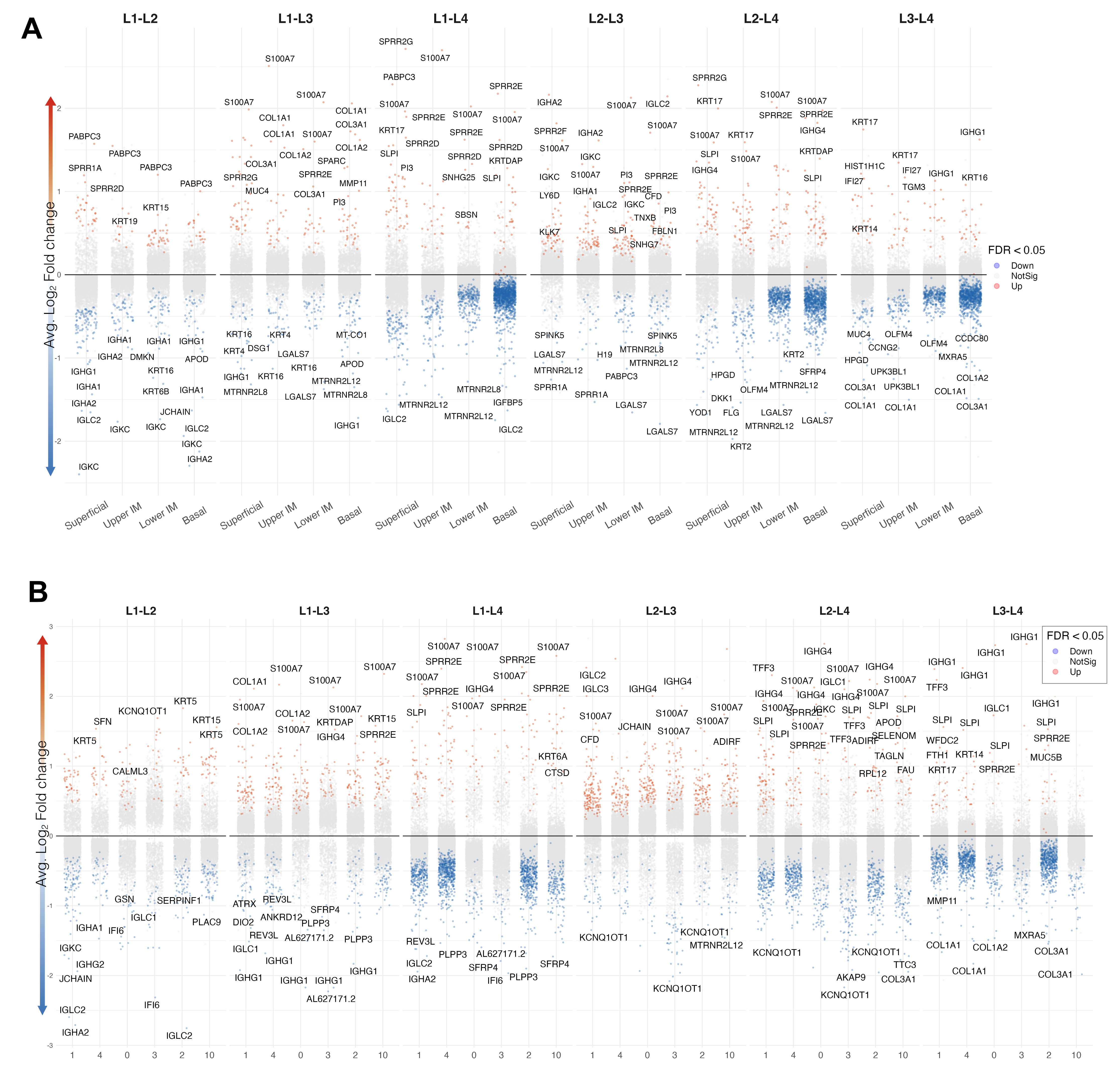

Supplement: S4 Fig — Volcano plots of all A epithelial and B submucosal DEGs for the Wilcox pairwise model, for each cluster on the x-axis and log fold change on the y-axis. (TIFF) [file ppat.1013677.s004.tiff]

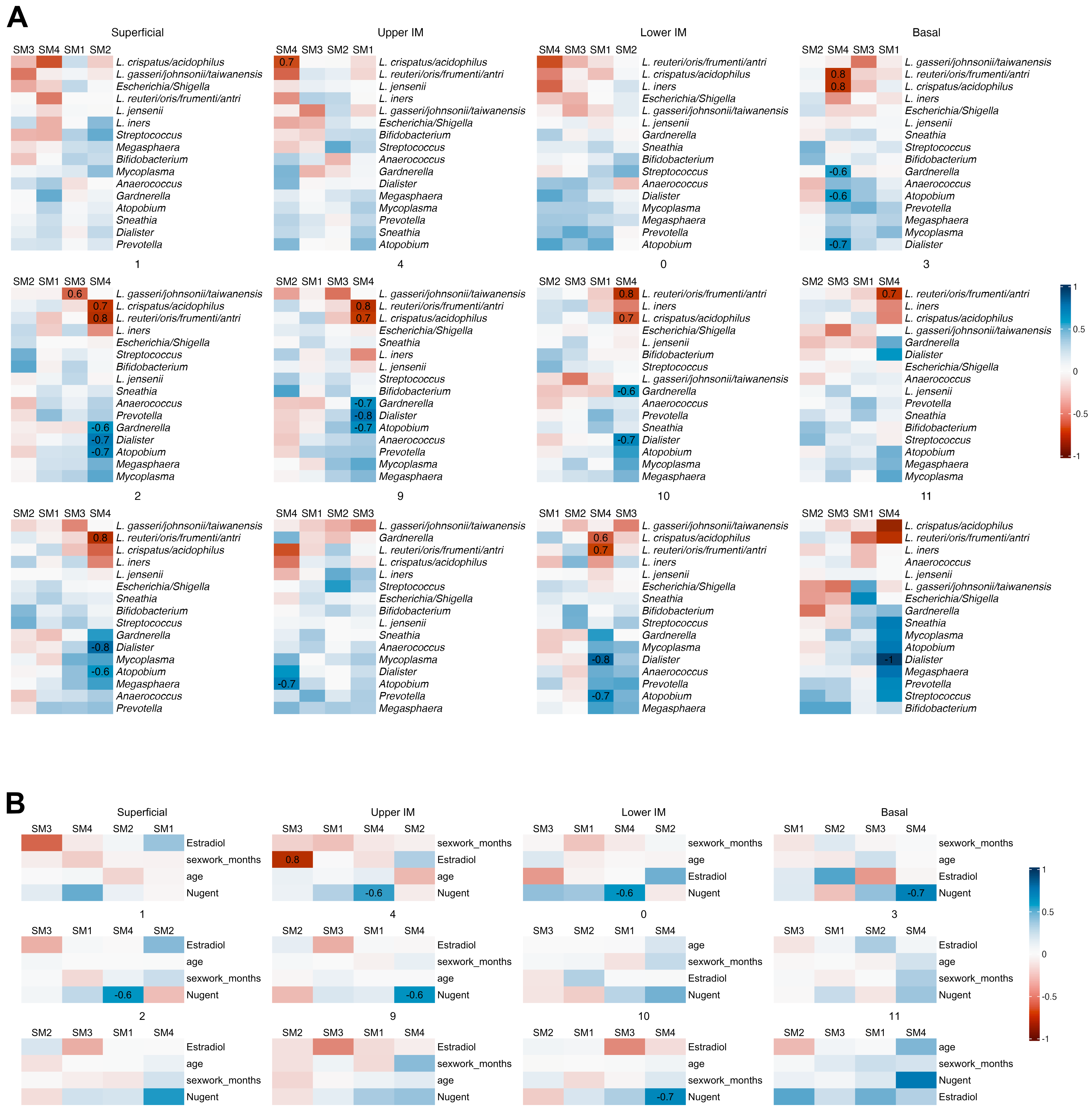

Supplement: S5 Fig — Heatmaps displaying Spearman’s correlation between gene modules and A 16 of the most abundant taxa for all clusters or B clinical variables of interest. The numbers indicate significant rho values after FDR adjustment. (TIFF) [file ppat.1013677.s005.tiff]
