# Supplementary material for: The cervicovaginal microbiome associates with spatially restricted host transcriptional signatures throughout the human ectocervical epithelium and submucosa
Source: PLoS Pathog. 2025 Nov 11;21(11):e1013677. doi: 10.1371/journal.ppat.1013677 (PMC12626330; doi:10.1371/journal.ppat.1013677)
Supplement: S1 Text — The rationale for combining pseudo-bulk and spot-level (Wilcoxon) approaches, as well as the steps taken to minimize biases such as pseudo-replication and inflated p-values, are explained in detail. Additional information is also provided on quality control, filtering thresholds, and the criteria used to define significantly differentially expressed genes (DEGs). (PDF) [file ppat.1013677.s013.pdf]

Supplementary Methods

Identification of microbiome-associated patterns by differential gene expression

Identification of microbiome-associated patterns by differential gene expression was performed by three methods to ensure a comprehensive analysis while minimizing biases. These methods included pseudo-bulk differential gene expression: (i) across the L1-L4 groups, revealing 709 differentially expressed genes (DEGs, FDR < 0.05), and (ii) pseudo-bulk pairwise across all group combinations revealing 1,346 DEGs, and lastly, (iii) a Wilcox pairwise study comparison resulting in 10,947 DEGs (**S2-S7 Table**). Pseudo-bulk analysis involves aggregating raw expression values (e.g., by summing or averaging) across cells or spots within each group, enabling the use of conventional bulk RNA-seq tools and addressing the issue of pseudo replication common in spatial data. In contrast, the Wilcoxon rank-sum test is applied at the individual spot level, which, while more sensitive, can lead to inflated p-values due to treating spatially proximate spots as independent. Method (i) involved a global comparison across all four groups simultaneously, analogous to a Kruskal–Wallis test, allowing for the identification of genes that vary across the full spectrum of group identities. Method (ii) and (iii) focused on all pairwise group comparisons (e.g., L1 vs. L2, L1 vs. L3, etc.), which helps to uncover more specific DEGs that may not reach significance in a global test due to variability confined to certain pairs. Overall, there was significant overlap in the definition of DEGs between all three computational approaches. Only nine genes from the pseudo bulk “across” analysis did not overlap with any of the two other methods (**Fig 2A**).

Wilcox pairwise comparisons revealed that the highly diverse (L4) group exhibited the most substantial alterations in gene expression. This group consistently showed the strongest transcriptional differences relative to the other three microbiome profiles, highlighting its distinct biological signature. Based on this observation, we focused our attention on the three pairwise comparisons that include the L4 group, as these provided the clearest insights into microbiome-associated gene expression changes. Accordingly, the following two sections present differential gene expression results specifically from these L4-involving comparisons. The results of the L1-L2, L1-L3, and L2-L3 comparison is available in supplementary tables (**S5–S7 Table**). Furthermore, although several ribosomal and mitochondrial genes were differentially expressed between the study groups, we focused our analysis on nuclear-encoded genes with known or putative functional relevance. This decision was made to avoid potential confounding from housekeeping or stress-related gene expression and to prioritize biologically interpretable signals.
