# Supplementary material for: The cervicovaginal microbiome associates with spatially restricted host transcriptional signatures throughout the human ectocervical epithelium and submucosa
Source: PLoS Pathog. 2025 Nov 11;21(11):e1013677. doi: 10.1371/journal.ppat.1013677 (PMC12626330; doi:10.1371/journal.ppat.1013677)
Supplement: Attachment — Submitted filename: Response to Reviewers.docx [file ppat.1013677.s014.docx]

Author’s responses (in bold) to reviewer’s comments

The line numbers refer to word document with tracked changes. NB! they are not consistent with the clean copy version.

We would like to inform the editors that one supplementary figure had not previously been referenced in the text. This has now been corrected, and the order of all supplementary figures has been revised to ensure consistency with the text.

**Part II – Major Issues:**

Reviewer #1:

I do not believe additional experiments are required to validate the study’s conclusions. However, I do recommend substantial revisions to the language used in interpreting the data. The manuscript frequently implies causality between microbiome composition and host gene expression, despite being based on cross-sectional data. To ensure the conclusions are appropriately supported, the authors should revise the discussion and results sections to consistently reflect associative relationships rather than mechanistic ones. This change is essential for maintaining interpretive rigor, especially in a field where microbiome findings often inform clinical or public health strategies.

**We have now carefully revised the Title, Abstract, Results, and Discussion sections to ensure that the language reflects associations rather than implying causality.
Revisions have been done at the following line numbers (the numbers refer to the manuscript version with tracked changes):**

**Title: Line 1**

**Abstract: Line 51 and 59**

**Author Summary: 71**

**Results: Lines 262, 367-368**

**Discussion: Lines 399-408, 434, 451, 452, 491, 492, 508**

Reviewer #2:

(No Response)

Reviewer #3:

1. First, the 10x Genomics Visium platform’s 55 µm spot size precludes single-cell resolution, with substantial gaps between spots; while this is an intrinsic limitation of the technology at the time of the study, it warrants more explicit discussion in the context of interpreting spatial specificity.

**We agree with the reviewer that the spacing between spots represents an important limitation that warrants explicit acknowledgement. We have now added a statement to the Discussion (lines 517-519). The text clarify that the spatial specificity of our findings should be interpreted at a regional rather than single-cell level. Although this constraint is inherent to the technology available at the time of our study, the consistent transcriptional patterns observed across clusters and layers support the robustness of our conclusions and reduce the likelihood that they are driven by spot-level artifacts.**

2. The use of unsupervised clustering without mapping to single-cell reference datasets led to mixed-cell type clusters (e.g., containing both B-cell and keratinocyte markers), which could complicate biological interpretation.

**We agree that unsupervised clustering on Visium data does not achieve pure cell-type resolution and will result in mixed-cell type clusters (e.g., B cell and keratinocyte markers within the same cluster). While deconvolution with a single-cell reference dataset could in principle help map transcripts to specific cell types, in our experience this approach is only reliable when a true scRNA-seq reference generated from the same cohort is available. In the absence of such a reference, we chose not to perform deconvolution, as we would not fully trust the results at the single-cell level. Instead, we focused our analyses on consistent transcriptional patterns observed across epithelial layers and submucosal clusters, rather than attributing expression to individual cell types. See addition to previous review response (reviewer no 3, point no 1).**

3. Finally, the causal direction between microbiome composition and host gene expression remains unresolved; the data cannot exclude the possibility that host transcriptional states influence microbiome structure rather than vice versa. There is no discussion of this fact or suggestions of future directions to elucidate this.

**This is an important point which has now been added to the Discussion (line 498-499). Please also see above for the revised language regarding causality/associations as specified in “Part II: Reviewer #1”.**

**Part III – Minor Issues: Editorial and Data Presentation Modifications**

Reviewer #1:

Introduction:
Line 98: Requires reference. “An optimal cervicovaginal microbiome is typically dominated by Lactobacillus species, which help maintain an acidic environment that protects against genital infections.”

**We have now added HIV as an example (line 99), which we think is a striking example, as well as a reference (Gosmann et al. 2017, https://doi.org/10.1016/j.immuni.2016.12.013)**

Line 100: Requires reference. “However, an anaerobic, highly diverse microbiome dominated by non-Lactobacillus bacteria is more commonly observed in women in sub-Saharan Africa.”

**We have now added a reference which is a seminal report on this topic**

**(Ravel et al. 2011, https://doi.org/10.1073/pnas.1002611107)**

Lines 118-125: The end of the introduction refers to a prior study as the basis for this manuscript, making the current text inaccessible to naïve readers. I recommend adding 1-2 sentences briefly outlining the studies referenced to set up the study design of this manuscript (cross-sectional or longitudinal).

**This clarification has now been added to these lines (121-124) in the Introduction.**

Results:
Line 131: Add space. “The previously published16S rRNA V4 gene sequencing data”

**This is now the beginning of its own paragraph (line 138)**

Figure S1: Is there a way to indicate on the figure to which study group (L1-L4) the samples belong?

**This has now been addressed. The updated figure now indicates the study groups (L1–L4) for each sample. Please note that the figure has been renumbered to S2 due to a previous error in the ordering of the supplementary tables.**

Lines 147-289: Across this section of results, I broadly recommend altering the topic sentence of each paragraph with the “conclusive” statement currently found at the end of each paragraph. Such a strategy would improve contextualizing the specific results reported. For example, in 276-288, instead of the current topic sentence “We next investigated the remaining pairwise group comparisons (Table S5–7)”, replace this with “We observed tissue-wide downregulation of antibody genes in the L. iners (L2) compared to the L. crispatus (L1) group (Table S5–7)”.

**Thank you for this helpful suggestion. We agree that moving the conclusive statements to the beginning of the paragraphs improves clarity and contextualization of the results. We have now updated the topic sentences in two places (line 205 and 298) where this restructuring made the most sense and enhances the flow of the section.**

Line 158-159: “Top marker genes” is vague. I recommend updating the sentence to “The top marker genes for each cluster, identified through one-vs-rest differential expression analysis, largely aligned with their spatial location within the tissue architecture”. Also, I think the reference to Fig. 1B should be replaced with “Fig. 1C, top panel”.
**Line 165 is now updated with the suggested text.**

Table S1: Please add this to the table caption the comparisons being made resulting in logFC values? For example, for the first gene, CRCT1, what is the comparison resulting in the reported increased expression (logFC=3.24)?

**… “vs rest” have now been added to the caption for each sheet.**

Lines 167-168: I suggest updating this sentence with: “The gene expression profiles of the submucosal clusters were less discernable and displayed a less structured spatial distribution compared to the epithelial layers (Fig. 1C, lower panel; Table S2)”
**Line 177-178 is now updated with the suggested text.**

Figure 1D: What are the units of the average expression indicated in the legend?

**The values represent *log-normalized expression* as computed by Seurat’s NormalizeData() function (default method = LogNormalize). Specifically, gene counts are normalized by the total counts per spot, scaled to 10,000, and log-transformed (log1p). The average values shown in the plot are therefore unitless and reflect the mean log-normalized expression across cells.**

**We have now added to the figure legend 1D:
The average values reflect the mean log-normalized expression across cells.**

Figure 2C: It may be helpful to indicate the layer in which the gene is enriched.

**The plotted values reflect normalized expression values averaged across the Superficial, Upper Intermediate, Lower Intermediate and Basal clusters. This has now been added to the figure legend.**

Lines 206-218 & Figure 2C: The text would be better supported by Figure 2C if there were an indication of the epithelial layer (stratify by or annotate with epithelial layer). Also, regarding the IG genes, my examination of the data suggest that the Upper IM actually has a “broader range” of upregulated genes (see figure below).

**As stated above for Figure 2C, the values are averaged across the epithelial layers. Thus, they will not always reflect the exact examples mentioned in the text.**

**Thank you for pointing out that it should be the Upper Intermediate and not the Lower. We have now corrected this mistake!**

Lines 220-233: OLFM4 is downregulated by less than 1-fold in each comparison. While the association is strong, I wouldn’t call the level of downregulation strong. Also, the text is a bit scattered after Line 222 (the first sentence). I appreciate that these data are multi-faceted and complex reflecting the nuanced relationships between the host tissue and exposure to the VMB. This is why a well-structured and flowing narrative is especially essential. As is, it is more like a list of findings. For example, KLK and KRT genes are referred to again, though they were mentioned two paragraphs up.

**In our data the significant comparisons of OLFM4 shows log2FC values between –0.33 and –1.29, which translates to approximately a 1.3- to 2.5-fold decrease in expression across comparisons. The interpretation of fold change from log2-transformed values can sometimes be unintuitive, as fold change values are asymmetric around 1. For example, a log2FC of +1 corresponds to a 2-fold increase in expression, while a log2FC of –1 corresponds to a 0.5-fold change (a 2-fold decrease in expression). While this does represent consistent downregulation, we agree with the reviewer that the magnitude is modest. To better reflect this, we have removed the word “strongly” in our description (line 244).**

**We do agree with the reviewer that it is confusing to reintroducing the KLK and KRT genes in the downregulation paragraph, since these genes had already been discussed in the upregulation context earlier. To improve clarity and flow, we have now moved this text to the upregulation paragraph (lines 228-230), thereby avoiding redundancy and ensuring that each section maintains a more focused and structured discussion.**

Supplemental Tables. Each of Tables S2-S7 have the epithelial layer and cluster results separated across multiple tabs. The layer and cluster data could each be easily combined into a single tab because the “layers” column indicates already the layer or cluster. This would make these data much easier to explore for readers.

**We appreciate that combining all clusters and layers into a single tab could make the data easier to explore for some readers. However, we believe that the current multi-sheet layout provides a clearer and more manageable structure for the general reader. For those interested in more advanced analyses, the data can easily be programmatically recombined in R or similar tools, allowing for a more in-depth investigation while keeping the supplemental tables accessible and organized.**

Figure 3B. As in Figure 2C, this would benefit from stratification by or annotation with the submucosa clusters in which the genes were enriched.

**As stated in the figure legend, the values on the y-axis are averaged across cluster 1, 4 and 2, which is why there is no submucosal cluster label on the plot.**

Line 293: This text and intro mention 3,771 DEGs, but the sum of “1,403, 1,078, and 1,041” (Line 202) is 3,522.

**The numbers you refer to are the epithelial genes only. The WGCNA analysis also contains the submucosal DEGs.**

Figure 6A: To improve interpretation, I recommend adding the brief description of each SM to above the SM label (at an angle for space) in the correlation plots. For example, “SM1: epithelial barrier function”. Also, please italicize row labels of genus and species names. Atopobium is now Fannyhessea. It is strange that Basal, 1, 10, and 2 were specifically plotted… Seems it would be clearer to use either layers or clusters as the facets here.

SM1 (summarized as ‘epithelial barrier function’)
SM2 (‘protein production’)
SM3 genes (‘inflammation and processes occurring in the extracellular space’)
SM4 genes (‘regulation of intracellular protein transport and RNA processing’)

**We have now added this text as a legend below the figure.**

Line 332: The statement “The data confirm the microbiome-associated altered gene expression” implies a level of causality that is not supported by the study’s cross-sectional design. As the authors have not performed longitudinal or mechanistic experiments, the findings should be interpreted as associative rather than causal. This distinction is particularly important in microbiome research, where observational data are often used to inform clinical or public health strategies. In the context of HIV susceptibility, overstating causality may mislead readers or policymakers. I recommend revising this phrasing to reflect the correlational nature of the findings—for example, “The data reveal microbiome-associated differences in gene expression” or “The data are consistent with microbiome-associated transcriptional variation.”

**We have now revised the text as suggested. Please also see above for the revised language regarding causality/associations as specified in “Part II: Reviewer #1”.**

Lines 341-343: Where are the data to support this? Figure 6A has plots for only Basal, 1, 10, and 2.
Lines 347-349: Where are the data to support these statements about relationships with nugent scores, age, sex work?

**We have now added a supplemental figure S5 to support this text.**

Throughout the discussion section, several statements imply a causal relationship between microbiome composition and host gene expression or tissue-level changes. Given the cross-sectional and observational nature of the study, the data support associations rather than causality. Phrases such as “responses,” “related to,” or “confirm” suggest mechanistic links that cannot be established without longitudinal or experimental validation. I recommend revising the discussion to consistently reflect the associative nature of the findings. This distinction is particularly important in microbiome research, where interpretations often inform clinical or public health strategies, and in the context of HIV susceptibility, where mechanistic clarity is critical. Some examples:
• “we were able to identify the spatial localization of these responses”
• “A highly diverse microbiome exhibited the largest differences in gene expression”
• “differences are related to”

**We have now revised the text as suggested. Please also see above for the revised language regarding causality/associations as specified in “Part II: Reviewer #1”.**

Lines 361-363: The sentence “A highly diverse microbiome exhibited the largest differences in gene expression compared with microbiome groups dominated by L. crispatus, L. iners, and Gardnerella taxa” is both unspecific and potentially misleading. It is unclear whether “gene expression” refers to changes across the entire tissue, specific epithelial layers, or submucosal clusters. Given the spatial resolution of the study, I recommend specifying the anatomical or transcriptional compartments where these differences were most pronounced.

**We agree with the reviewer that the original wording was too general and did not reflect the spatial dimension of our findings. We have therefore revised the sentence to specify the anatomical compartments where the strongest transcriptional differences were observed.**

**Updated text (Lines 399–402):**

**“The highly diverse microbiome group was associated with the most pronounced differences in gene expression, primarily located around the epithelial membrane, compared to microbiome groups dominated by *L. crispatus*, *L. iners*, and *Gardnerella* taxa.”**

Discussion:
Lines 356–447: Throughout the discussion section, several statements imply a causal relationship between microbiome composition and host gene expression or tissue-level changes. Given the cross-sectional and observational nature of the study, the data support associations rather than causality. Phrases such as “responses,” “related to,” or “confirm” suggest mechanistic links that cannot be established without longitudinal or experimental validation. I recommend revising the discussion to consistently reflect the associative nature of the findings. This distinction is particularly important in microbiome research, where interpretations often inform clinical or public health strategies, and in the context of HIV susceptibility, where mechanistic clarity is critical.

Some examples:

- “we were able to identify the spatial localization of these responses” (line 359)
- “A highly diverse microbiome exhibited the largest differences in gene expression” (line 361)
- “These differences are related to disruption of epithelial and ECM structures” (line 363)
- “The data confirm the microbiome-associated altered gene expression” (line 331)
- “ the most predominant gene expression alterations” (line 387, and “alterations” in line 389). Changes were not measures, differences were.
- “We could also confirm and now spatially localize previously presented findings, including microbiome-associated alterations of immune-related mechanisms that can facilitate HIV penetration (7,32,33) and trigger recruitment of activated CD4+ T cells to the genital mucosa, promoting HIV spread (7,34,35). (lines 440-444).

**We have now revised the text as suggested. Please also see above for the revised language regarding causality/associations as specified in “Part II: Reviewer #1”.**

Lines 365–367: The interpretation of “homeostatic immune activity” and “mucosal barrier integrity” in the L. crispatus group is speculative, as it is based solely on transcriptomic data. These biological states typically require protein-level or functional validation. I recommend softening the language to reflect inferred transcriptional signatures rather than confirmed immune or structural states.

**We agree with the reviewer and have now revised the lines accordingly (lines 404-408).**

Lines 379–381: The sentence “It is therefore surprising that our data indicated activated host responses throughout the entire ectocervical mucosa, which we interpreted as molecular signals driven by the cervicovaginal microbiome” overstates the novelty of the finding. Prior studies (e.g., Blaskewicz et al., 2011; Arnold et al., 2015; Anahtar et al., 2015) have shown that the apical ectocervical epithelium is permeable to macromolecules such as IgG and that microbial communities can influence immune activation beyond the luminal surface. I recommend revising this sentence to reflect that the observed transcriptional changes are consistent with known mucosal biology and to avoid implying causality.

**Thank you for this relevant comment. While keeping the reference to Blaskewicz et al., 2011 (ref. 21 in the original version), we hesitate to refer to Arnold et al, 2015, and Anahtar et al, 2015, in this context. Those latter two studies did not assess cervical tissue biopsies and can thus not be compared to our present study applying spatial transcriptomics on tissue biopsy samples. Nevertheless, we have carefully revised the sentence to hopefully capture the reviewer’s intention (lines 420-422).**

Lines 392–423: The discussion of IgA and IgG gene expression (lines 411–423) could be better anchored in mucosal immunology literature. Torcia (2019) and Takada (2025) provide broader context for how IgA and IgG contribute to microbial clearance and immune tolerance in the cervicovaginal mucosa. Including these references would help clarify the implications of the observed transcriptional differences and better connect them to established host-microbiome interaction mechanisms.

Lines 411–423: The discussion of IgA and IgG expression and their roles in microbial modulation is informative but could be better integrated with known mucosal immunology. Consider elaborating on how these findings relate to established mechanisms of immune exclusion or tolerance in the cervicovaginal mucosa.

**Thank you for these comments. New data demonstrating IgG and IgA subclass gene expression differences across microbiome groups have now been added in the Results section (lines 316-324), along with a new figure, Fig. 4B. A new Discussion section on these Ig subclasses, their potential mechanisms, as well as new references (including Torcia (2019) and Takeda (2025)), have now been added to the manuscript (lines 467-482). This Discussion section replaces the previous one which was less clear.**

Lines 438–439: The phrase “tissue-wide impact” should be clarified. The data show that transcriptional changes are most pronounced near the basal membrane and in specific submucosal clusters. Please specify which compartments are affected to better reflect the spatial resolution of the findings.

**We agree with the reviewer that the phrase “tissue-wide impact” was too general and did not adequately reflect the spatial resolution of our findings. We have revised the text to specify the affected compartments more precisely. See revised sentence at line 500-503.**

Reviewer #2:

- It would be helpful to address limitations of the study in the discussion. For example, the inability to infer directionality of the microbial-host transcriptional associations, transcriptional findings were not further validated through other methods (i.e. protein quantification, IHC), and fungal and viral associations were not addressed, etc.

**Thank you for pointing this out. We have now added a new section to the discussion (lines 515-534) highlighting the major limitations of the study.**

- Consider rewording the title. The study identifies transcriptional associations with vaginal bacterial composition and the directionality of these associations can not be inferred from the data – thus the microbiome may not impact host transcriptional signatures.

**The Title has been updated accordingly.**

- Consider de-emphasizing the conclusions related to susceptibility to HIV acquisition. This was not the focus of the study, and all participants were HIV negative.

**We appreciate the reviewer’s point regarding HIV susceptibility. While all participants in our study were HIV negative, this remains a highly susceptible cohort. For example, some cohorts of sex workers have been shown to have a 21-fold greater risk of HIV infection compared to women in the general population. We therefore believe that discussing our findings in the context of HIV susceptibility is relevant and provides important biological insight, even if susceptibility itself was not directly measured in this study. For clarity, we have ensured that our conclusions remain focused on host–microbiome interactions while contextualizing their potential relevance for HIV acquisition risk.**

- The text mentions L. crispatus or L. jensenii in group 1 – Fig. 1A indicates L. crispatus/acidophilus. Please clarify and check for congruency with Supp. Fig. 4.

**We have now replaced L. crispatus with L. crispatus/acidophilus in the Abstract and at the first mention in the Results section to ensure consistency with Fig. 1A and Supplementary Fig. 4. In subsequent mentions, we continue to use the simplified term “L. crispatus” for readability.**

- Consider updating taxonomy where relevant. Atopobium is now referred to as Fannyhessya (PMID: 30186281)

**We thank the reviewer for this important note. The taxonomy used in our analyses is based on an earlier database version, which still lists Atopobium. For consistency with the underlying data, we have kept this nomenclature in the results. However, to avoid confusion, we have clarified at the first mention in the manuscript that Atopobium is now referred to as Fannyhessya.**

Reviewer #3:
Grammatical/formatting Issues:

1. Line 169: “Cluster 4 exhibited increased expression of several immunological and endothelial genes. Clusters 0 and 3 showed down regulation of genes that were more pronounced in other clusters.” Wording is vague and the second sentence is redundant with the idea of DEG. I think it can be combined with the following one and simplified.

**We thank the reviewer for this suggestion. We have revised the text to improve clarity and remove redundancy. The updated version now reads (lines 181-186):**

**“Cluster 4 showed elevated expression of immunological and endothelial genes, while Clusters 0 and 3 exhibited reduced expression of several genes, including C12orf57, which is ubiquitously expressed in human tissues. This suggests that these clusters represent areas with low cell density.”**

2. Lines 180- 184- have any previous studies done transcriptomics of these tissues? GEX or spatial? Would be good to mention

**We thank the reviewer for this helpful suggestion. As noted on lines 119–120, spatial transcriptomics has previously been applied to human cervical tissue in the context of HPV-associated cancers, hormonal contraceptive use, and HIV infection. We have now updated this section with two additional references to ensure completeness. Furthermore, we have revised the Results text (now lines 197–201) to explicitly highlight how our study extends beyond these earlier efforts by providing a more detailed map of healthy ectocervical tissue architecture.**

3. Line 216 ref: “…including MIF and FABP5, which are linked to macrophage responses and retinoic acid metabolism”

**References have now been added**

**https://doi.org/10.1016/j.celrep.2022.111668**

**https://doi.org/10.1038/ncomms9794**

4. Line 232 ref: “…STAT3 activator and is involved in tissue repair”

**A reference has now been added**
**https://doi.org/10.1038/s41568-018-0090-8**

5. Line 263 ref: “ involved in cell adhesion and ECM remodeling.”
**References have now been added**

**https://doi.org/10.1146/annurev.cb.11.110195.003001**

**https://doi.org/10.1016%2Fj.bmc.2007.01.011**

**https://doi.org/10.1074/jbc.M116.773713**

6. Lines 270-272: “ L1 was associated with upregulation of various innate immune and barrier function genes that maintain a protective and balanced immune environment”
a. It was not discussed previously in this section which genes upregulated in L1 are associated with barrier function and no reference to how they’ve been shown to maintain protective and balanced immune environment

**We thank the reviewer for this helpful comment. To improve clarity, we have now specified which genes we are referring to in this sentence. The revised text reads:
“L1 was associated with upregulation of various innate immune and barrier function genes (e.g., KLK, SPRRR, S100) that maintain a protective and balanced immune environment [REF].”
We have also added the appropriate reference regarding the role of these genes in the cornified envelope and barrier function (Candi, et al, 2005, https://doi.org/10.1038/nrm1619).**

7. No comment on how L4 was more distinct when looking at pairwise comparisons of DEG, but L3 stood out when analyzing the spatial gene modules

**We believe that the reviewer refers to SM1 and SM2 where L3 stands out in selected layers. Such a discrepancy is not surprising since DEG analysis identifies individual genes that show statistically significant changes in expression between conditions. As a complement, WGCNA explores patterns of gene co-expression to identify modules of genes that work together, offering a more systems-level view of biological processes.**

8. Line 349 what is a Nugent score?

**A short explanation has now been added at line 385-386.**

9. Line 418 ref: “…facilitate bacterial clearance and immune tolerance.
10. Line 419 ref: “… IgG promotes death and clearance of bacteria through complement…”

**These lines have been replaced by a new section.**

11. Line 448-450. This is a poor final sentence to end the discussion section as it is introducing an example rather than summarizing final conclusions or future directions.

**A new paragraph has now been added talking about future directions.**
