# Supplementary material for: The cervicovaginal microbiome associates with spatially restricted host transcriptional signatures throughout the human ectocervical epithelium and submucosa
Source: PLoS Pathog. 2025 Nov 11;21(11):e1013677. doi: 10.1371/journal.ppat.1013677 (PMC12626330; doi:10.1371/journal.ppat.1013677)
Supplement: Attachment — Submitted filename: Response_to_Reviewers_auresp_2.docx [file ppat.1013677.s015.docx]

**Reviewer #1 Comment:**
Ensure supplemental figures and figure legend numbers match.

- Fig S1: Legend is “S1 Figure. Quality control,” but provided figure labeled as “Figure S1” is “16S taxonomical annotation.”
- For the “16S taxonomical annotation” supplemental figure and Fig. S5: species and genus names need to be italicized. The legend should be “16S rRNA gene-based proportions of vaginal taxa” or something to that effect.
- Figure 6: italicize species and genus labels.

**Response:**
We thank the reviewer for these helpful observations and have carefully reviewed all figure numbering, legends, and taxonomic formatting. The inconsistencies between the supplemental figure numbers and legends have now been corrected.

For the former “16S taxonomical annotation” figure, the legend title has been updated to **“Figure S1. 16S rRNA gene–based proportions and community structure of vaginal taxa”**, as suggested by the reviewer. In addition, all species and genus names in this figure, in Figure S5, and in Figure 6 have been italicized to be consistent with scientific nomenclature conventions.
